# Complete Resolution of a Fusobacterium necrophorum Liver Abscess With Short-Course Antibiotics and Drainage

**DOI:** 10.7759/cureus.108820

**Published:** 2026-05-13

**Authors:** Fatma Al Farsi, Maya Al Salti, Aisha Al Balushi, Mustafa Al Khaifi, Sultan Al Lawati

**Affiliations:** 1 Medical Laboratory, Rustaq Hospital, Rustaq, OMN; 2 Radiology, Rustaq Hospital, Rustaq, OMN; 3 Internal Medicine, Rustaq Hospital, Rustaq, OMN

**Keywords:** 16s rrna sequencing, culture-negative, diabetes mellitus, fusobacterium necrophorum, pyogenic liver abscess

## Abstract

A pyogenic liver abscess results from bacterial invasion of hepatic parenchyma via hematogenous, biliary, or direct routes. While *Enterobacterales *predominate, anaerobes such as *Fusobacterium necrophorum* are rare causes, often missed by conventional cultures due to prior antibiotics and fastidious growth. A 52-year-old man with diabetes, hypertension, and dyslipidemia presented with two weeks history of fever and epigastric pain. Labs showed leukocytosis (WBC: 12.22 × 10^9^/L, neutrophils: 8.91 × 10^9^/L) and CRP of 289 mg/L. A CT scan revealed a ring-enhancing abscess in segment IV measuring 4.9 × 3.5 × 4.9 cm. Drainage yielded 30 mL of pus. Ceftriaxone was started empirically, which was upgraded later to piperacillin-tazobactam. Blood and pus cultures were negative. 16S rRNA sequencing later identified *F. necrophorum* (99.79% match). He completed eight days of intravenous antibiotics but did not adhere to the prescribed oral antibiotic regimen after discharge. At the five‑month follow‑up, he demonstrated complete clinical and radiological resolution of the liver abscess. This case highlights *F. necrophorum* as an underdiagnosed cause of pyogenic liver abscess in patients with diabetes. Molecular methods, such as 16S rRNA sequencing, facilitate accurate pathogen detection and guide targeted antimicrobial therapy. In this patient, source control through drainage was sufficient even with a short course of antibiotics, suggesting that treatment duration may be individualized in selected cases.

## Introduction

A liver abscess results from the invasion and replication of pathogenic microorganisms that reach the hepatic parenchyma via hematogenous dissemination, direct extension from adjacent tissue injury, or translocation through the biliary tract [1]. Diabetes mellitus is recognized as one of the well-documented risk factors for a pyogenic liver abscess (PLA) [2]. *Enterobacterales* were the predominant pathogens in PLA, with enterococci and streptococci also frequently detected [3]. *Fusobacterium necrophorum *is a rare cause of PLA. It is most associated with Lemierre's syndrome. In this report, we describe a case of PLA attributable to *F. necrophorum*, in which the organism failed to grow on conventional culture but was subsequently identified through molecular diagnostic methods.

## Case presentation

A 52-year-old man presented to the Emergency Department with a two-week history of fever and epigastric pain. He also reported constipation for five days but denied nausea, vomiting, respiratory symptoms, or urinary complaints. He had no recent travel or animal exposure. His medical history was significant for diabetes mellitus, hypertension, and dyslipidemia. Prior to presentation, he had been evaluated at a primary healthcare clinic, where he completed a five-day course of amoxicillin-clavulanic acid without clinical improvement.

On examination, the patient was hemodynamically stable and afebrile. Abdominal examination revealed a soft abdomen with localized epigastric tenderness. Laboratory studies showed leukocytosis (WBC: 12.22 × 10^9^/L) with neutrophilia (8.91 × 10^9^/L), lymphocytes of 2.15 × 10^9^/L, eosinophils of 0.11 × 10^9^/L, and markedly elevated CRP (289 mg/L). Liver function tests were within normal limits.

**Figure 1:**
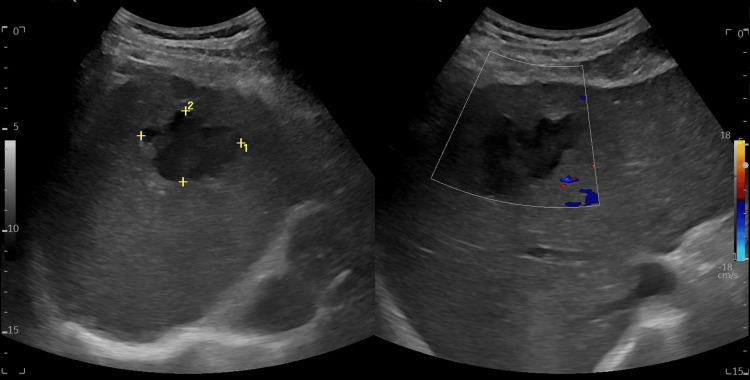
Ultrasound images showing a heterogeneous hypoechoic lesion in the right hepatic lobe with internal echoes and absence of posterior acoustic enhancement, without internal vascularity on Doppler, compatible with hepatic abscesses.

**Figure 2:**
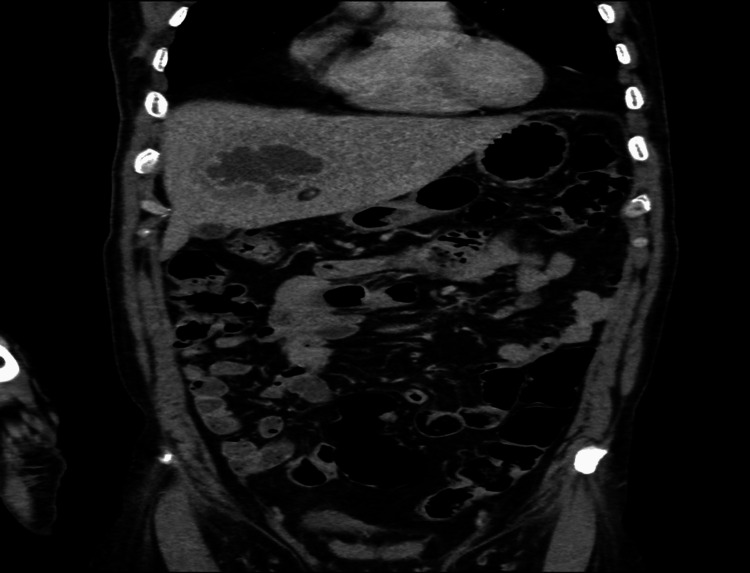
Contrast-enhanced CT imaging showing a well-defined hypodense lesion in the right hepatic lobe with central dense fluid attenuation and peripheral rim enhancement, consistent with hepatic abscesses.

He was admitted and started empirically on ceftriaxone while awaiting blood and urine culture results. On the third day of admission, he developed worsening epigastric pain. Abdominal ultrasound revealed a focal irregular hypoechoic lesion in segment IV of the liver, measuring 4.9 × 3.5 × 4.9 cm (Figure 1). A subsequent contrast-enhanced CT scan demonstrated a ring-enhancing lesion in the left hepatic lobe, highly suggestive of a hepatic abscess (Figure 2). His antibiotic regimen was escalated to piperacillin-tazobactam.

**Figure 3:**
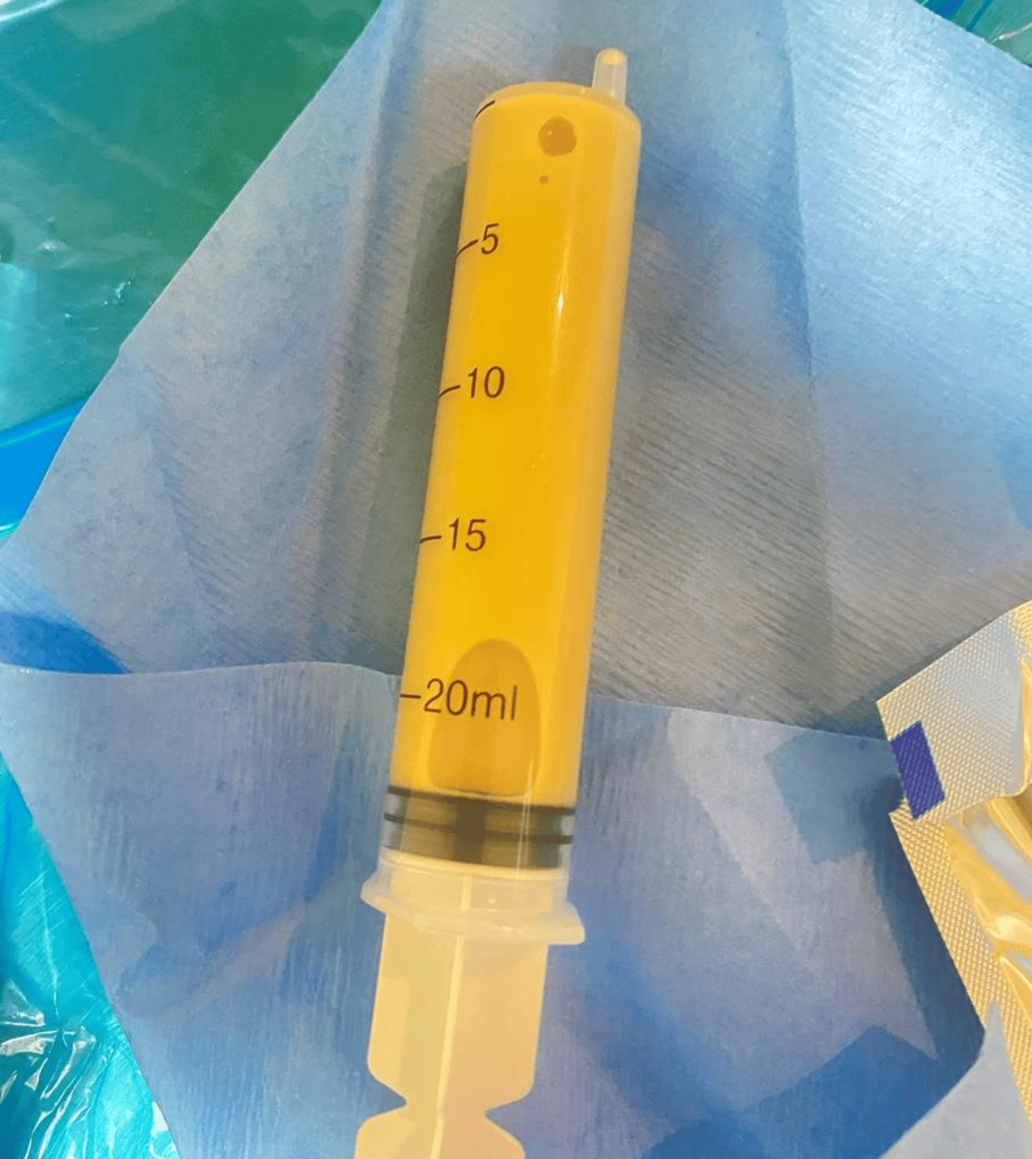
Aspirated liver abscess.

The patient underwent ultrasound-guided drainage of the liver abscess, yielding 30 mL of purulent fluid (Figure 3). Blood culture remained negative after five days of incubation, and abscess fluid cultures showed no aerobic or anaerobic bacterial growth after 48 hours. PCR testing for *Entamoeba histolytica *was negative, and the specimen was sent for 16S rRNA sequencing.

Clinically, the patient improved and was requested to continue treatment at home. He completed five days of piperacillin-tazobactam and was discharged on oral ciprofloxacin and metronidazole for two weeks. Two weeks later, 16S rRNA sequencing of the abscess fluid returned positive for *F. necrophorum*, with a matching score of 99.79%.

**Figure 4:**
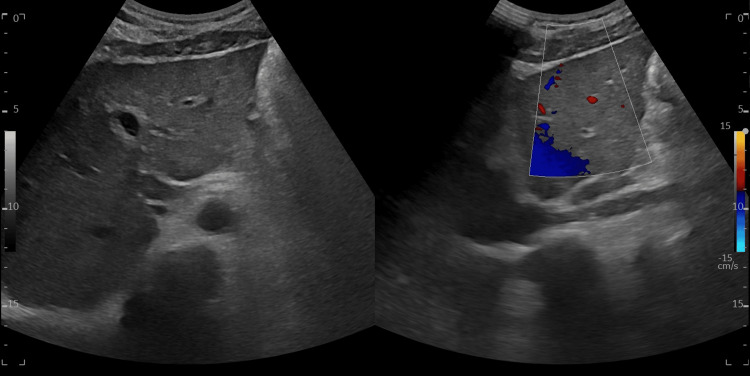
Follow-up liver ultrasound showing complete resolution of the liver abscess.

Five months later, he was contacted for follow-up. Surprisingly, he did not take any antibiotics after discharge and missed his follow-up appointment. He was completely well and asymptomatic; laboratory tests showed a normalized CRP (down from 303 to < 5 mg/L), and abdominal ultrasound confirmed complete resolution of the liver abscess (Figure 4).

## Discussion

A liver abscess is a serious, potentially life-threatening infection of the liver characterized by localized pus formation within hepatic parenchyma. The incidence of liver abscess has increased due to advanced diagnostic services [4]. The commonest risk factors for liver abscess comprise diabetes mellitus, immunosuppression, intra-abdominal diseases, and biliary surgery. The clinical presentation can be non-specific, and this might lead to late or missed diagnosis [5]. It can be a primary consequence of liver injury or secondary due to disseminated infection, mainly from intraabdominal compartment. It is categorized mainly as pyogenic or amoebic. While most amoebic liver abscesses are caused by the *E. histolytica*,* *the pyogenic abscesses vary geographically, with common pathogens being Enterobacteriaceae, mainly *Klebsiella pneumoniae* and *Escherichia coli,* *Staphylococcus aureus*, and *Streptococcus *species [6].

The anaerobic pathogens are less common, probably underdiagnosed due to difficulty in culture and identification, which poses a challenge in management [7]. Different anaerobic pathogens reported as causative of liver abscess, such as *Fusobacterium* species, *Bacteroides *species, *Prevotella oris, Porphyromonas endodontalis, *and *Parvimonas micra* [8,9].

*Fusobacterium *species is an anaerobic Gram-negative bacillus typically associated with Lemierre's syndrome or oropharyngeal infections. Both *F. nucleatum *and *F. necrophorum *have been increasingly recognized as important etiological agents of pyogenic liver abscess, especially in culture-negative cases, necessitate using advance molecular testing such as 16S rRNA sequencing [10].

Traditional microbiological testing, including blood and aspirate cultures, is frequently employed to diagnose liver abscesses, but it has certain drawbacks. These cultures generally take 48-72 hours and often have low success rates for pathogens that require specific cultivation conditions, such as anaerobes and amoebas, leading to a detection sensitivity of around 40% [11]. After antibiotic treatment, the culture positivity rate drops to 27.7% [12]. In this case, patients received empirical antibiotics before obtaining pus samples for culture; subsequently, aerobic and anaerobic cultures were unable to isolate *F. necrophorum*, which was later identified using 16S RNA. Without proper testing, this organism might remain undetected.

Clarridge highlighted the significance of advanced molecular techniques in accurately identifying bacterial pathogens that are culture-negative, thereby improving the precision of etiological diagnosis [13]. The 16S ribosomal RNA (16S rRNA) includes a "conserved" region found universally among bacteria and a hypervariable region that is specific for bacterial identification. Sequencing the 16S rRNA gene involves extracting DNA from clinical samples and using it as a template for PCR to amplify a segment of about 500 or 1500 bp of the 16S rRNA gene sequence. Bacteria can be identified by analyzing the nucleotide sequence of the PCR product and comparing it with known sequences in a database [13].

Numerous recent studies have utilized 16S rRNA sequencing for the prompt identification of unusual non-cultivable bacteria, facilitating the early initiation of definitive antibiotic therapy for liver abscesses. Bacteria identified using this method include *Eggerthella lenta, Aggregatibacter aphrophilus, Pannonibacter phragmitetus, Parvimonas micra, Streptococcus oralis, Fusobacterium *species*, Bacteroides *species*, Prevotella *species*, Peptostreptococcus,* unassigned Enterobacteriaceae, etc. [14-16]. However, 16S rRNA requires the acquisition of expensive equipment, ongoing maintenance of these platforms, development of specific laboratory infrastructure, training of technical expertise, and turnaround time, all of which remain challenges.

Generally, the management of a liver abscess requires both source control and prolonged antibiotic therapy. Needle aspiration is typically recommended for a single abscess smaller than 5 cm, while catheter drainage is preferred for larger lesions (> 5 cm). Surgical intervention is reserved for patients with multiple or loculated abscesses [17].

Most isolates of *F. necrophorum* are susceptible to several antibiotic classes. However, approximately 2% show resistance to penicillin, and 15% resistant to erythromycin [18]. Given that these infections are often polymicrobial, a combination of a third-generation cephalosporin with metronidazole is considered a safe and effective initial therapy [19].

The optimal duration of antibiotic therapy remains unclear, as no clinical trial has defined it precisely. Current practice suggests four to six weeks, guided by clinical response and follow-up imaging [20].

Our patient underwent abscess drainage and received a total duration of eight days of intravenous antibiotics (ceftriaxone for three days, piperacillin-tazobactam for five days, and metronidazole for four days). He showed clinical improvement after abscess drainage and a short duration of antibiotics.

## Conclusions

This case highlights *F. necrophorum *as a rare, underdiagnosed anaerobe in culture-negative pyogenic liver abscesses, particularly in diabetic patients, where prior antibiotics hinder conventional diagnosis. Advanced molecular tools such as 16S rRNA sequencing enable precise identification and optimization of targeted therapy. Effective source control drainage with a short intravenous antibiotic course can yield full resolution, advocating for individualized management based on clinical and radiographic response rather than rigid durations.
